# Diagnóstico clínico integral de adultos mayores atendidos en Perú*

**DOI:** 10.15649/cuidarte.2485

**Published:** 2022-10-22

**Authors:** José Ander Asenjo-Alarcón

**Affiliations:** 1 . Universidad Nacional Autónoma de Chota, Chota, Perú. Email: ander1213@hotmail.com Universidad Nacional Autónoma de Chota Universidad Nacional Autónoma de Chota Chota Peru ander1213@hotmail.com

**Keywords:** Diagnóstico Clínico, Práctica Integral de Atención, Salud del Anciano, Adulto Mayor, Datos Demográficos, Perú, Clinical Diagnosis, Integral Healthcare Practice, Health of the Elderly, Aged, Demographic Data, Peru, Diagnóstico Clínico, Prática Integral de Cuidados de Saúde, Saúde do Idoso, Idoso, Dados Demográficos, Peru

## Abstract

**Introducción:**

Los adultos mayores constituyen el grupo poblacional más vulnerable y el menos favorecido por los sistemas de salud, transitan por un proceso de involución progresiva que puede repercutir en su salud; por tanto, se torna relevante el diagnóstico clínico oportuno y adecuado para identificar eventuales alteraciones.

**Objetivo:**

Establecer el diagnóstico clínico integral según sexo y edad de adultos mayores atendidos a nivel nacional en el Perú.

**Materiales y métodos:**

Se realizó un estudio descriptivo, transversal y retrospectivo, durante marzo y abril del 2021, en 60698 adultos mayores atendidos en todos los departamentos del Perú. La valoración física, funcional, mental y social de los adultos mayores se realizó mediante la Historia Clínica de Atención Integral de Salud del Adulto Mayor y se interpretó con su guía técnica. Se describieron frecuencias absolutas, relativas, intervalos de confianza para proporciones al 95% y el chi cuadrado de homogeneidad.

**Resultados:**

El 49,4% de adultos mayores estaban enfermos, de los cuales el 50,8% fueron mujeres y el 47,4% varones, el 50,0% tenían de 60 a 69 años, el 50,6% de 70 a 79 años y el 46,1% de 80 a más años. Se encontraron diferencias estadísticamente significativas entre el diagnóstico clínico integral por sexo y edad (p = 0,000).

**Discusión:**

Independientemente del número de participantes en todos los contextos estudiados, al menos una enfermedad crónica coexiste medianamente en los adultos mayores, sin ser determinante el lugar donde viven, pues el proceso de envejecimiento solo puede ser distinto en su velocidad de progresión y en las condiciones en que se presenta.

**Conclusión:**

Los adultos mayores en su mayoría estaban enfermos, fueron mujeres y tenían de 70 a 79 años, las diferencias por sexo y edad en el diagnóstico clínico integral fueron significativas. Las intervenciones en etapas previas contribuirían de manera importante en un envejecimiento saludable.

## Abstract (en)

**Introduction:**

Older adults are the most vulnerable population group and the least favored by health systems. They go through a process of progressive involution that can have health repercussions; therefore, timely and adequate clinical diagnosis becomes relevant to identify possible alterations.

**Objective:**

To establish a comprehensive clinical diagnosis according to sex and age of older adults attended nationally in Peru.

**Materials and Methods:**

A descriptive, cross-sectional, and retrospective study was conducted between March and April 2021 on 60,698 older adults attended in all the departments of Peru. Physical, functional, mental, and social examination of the older adults was conducted using the Medical Record of Comprehensive Health Care for Older Adults, interpreted using its technical guide. Absolute and relative frequencies, 95% confidence intervals for proportions, and the chi-square test of homogeneity were described.

**Results:**

49.4% of older adults were ill, of which 50.8% were women and 47.4% were men; 50.0% were aged 60 to 69 years, 50.6% were aged 70 to 79 years, and 46.1% were aged 80 years and over. Statistically significant differences were found among comprehensive clinical diagnosis, sex, and age (p = 0.000).

**Discussion:**

Regardless of the number of participants in all the contexts studied, at least one chronic disease moderately coexists in older adults, without the place where they live being a determining factor, since the aging process can only be different in its speed of progression and in the conditions in which it occurs.

**Conclusion:**

Most of the older adults were ill, female, and aged 70 to 79 years; differences in sex and age in the comprehensive clinical diagnosis were significant. Interventions at earlier stages may contribute significantly to healthy aging.

## Resumo

**Introdujo:**

os idosos constituem o grupo populacional mais vulnerável e menos favorecido pelos sistemas de saúde, passam por um processo de involucáo progressiva que pode afetar sua saúde; portanto, o diagnóstico clínico oportuno e adequado torna-se relevante para identificar eventuais alteracóes.

**Objetivo:**

estabelecer o diagnóstico clínico integral segundo sexo e idade de idosos atendidos em nível nacional no Peru.

**Materiais e métodos:**

foi realizado um estudo descritivo, transversal e retrospectivo, durante marco e abril de 2021, em 60.698 idosos atendidos em todos os departamentos do Peru. A avaliacáo física, funcional, mental e social dos idosos foi realizada por meio da História Clínica da Atencáo Integral a Saúde do Idoso e foi interpretada com seu guia técnico. Foram descritas frequencias absolutas e relativas, intervalos de confianza para proporcóes a 95% e o qui-quadrado de homogeneidade.

**Resultados:**

49,4% dos idosos estavam doentes, sendo 50,8% mulheres e 47,4% homens, 50,0% tinham entre 60 e 69 anos, 50,6% tinham 70 a 79 anos e 46,1% de 80 a mais anos. Foram encontradas diferencas estatisticamente significativas entre o diagnóstico clínico abrangente por sexo e idade (p = 0,000).

**Discussáo:**

Independentemente do número de participantes em todos os contextos estudados, pelo menos uma doenca crónica coexiste moderadamente em adultos mais velhos, independentemente do local onde vivem, uma vez que o processo de envelhecimento só pode diferir na sua velocidade de progressáo e nas condicóes em que ocorre.

**Conclusáo:**

os idosos eram em sua maioria doentes, eram mulheres e tinham de 70 a 79 anos, as diferencas por sexo e idade no diagnóstico clínico integral foram significativas. As intervencóes em etapas anteriores contribuiriam significativamente para um envelhecimento saudável.

## Introducción

La salud de los adultos mayores se ha visto mermada a través de los años, ya sea por influencia de sus condiciones inherentes o por factores externos como la práctica acumulada de estilos de vida poco saludables, factores ambientales o sociales, aspectos que inciden directamente en su nivel de fragilidad frente a cuadros patológicos y en su respuesta fisiológica a los mismos, alterando su calidad de vida en la última etapa^1^.

Es así que, el rendimiento físico y funcional de este grupo poblacional se compromete seriamente cuando presentan multimorbilidad, pues la acumulación de alteraciones orgánicas complejas generalmente crónicas limita la realización de actividades habituales y restringen las actividades que requieren de esfuerzo físico^2^. Entre las principales enfermedades crónicas no transmisibles que afectan a este grupo etario están las enfermedades cardiovasculares (provocadas esencialmente por la hipertensión arterial), diversos tipos de cáncer, enfermedades respiratorias y diabetes mellitus (generalmente la tipo 2), así mismo varias de estas enfermedades se presentan de forma conjunta, es decir que la afectación es simultánea en diferentes sistemas orgánicos, lo que agrava la situación, por la polifarmacia y las múltiples intervenciones que se tienen que realizar, dificultando así el manejo adecuado de éstas^3^. Por otro lado, las multimorbilidades limitan la movilidad y el funcionamiento físico suficiente en los adultos mayores, acentuando su grado de dependencia^4^.

Esta realidad es común en diferentes ámbitos geográficos, pues estudios realizados en Asia y América, reportan que cerca de la mitad de su población adulta mayor presentan enfermedades crónicas concomitantes, en China^5^ el 42,4% de adultos mayores padecía de más de una enfermedad, con mayor frecuencia en mujeres (p < 0,05), en la India^6^ y Nepal^7^ las cifras fueron superiores, con el 48,8% y 48,9% de afectados con una o varias enfermedades, las mujeres fueron las más proclives a padecerlas con el 50,4% y 52,4%, respectivamente. En Brasil^8^, más de uno de cada dos adultos mayores (53,1%), presentaban tres o más enfermedades crónicas, siendo característico en mujeres y en edades avanzadas.

Las enfermedades crónicas y las restricciones en la realización de actividades físicas como consecuencia, podrían repercutir considerablemente en la salud mental de los adultos mayores, provocando desequilibrios en sus capacidades superiores como la concentración, atención, aprendizaje, cognición, memoria, inteligencia y control emocional; puesto que las evidencias indican una asociación positiva entre los niveles adecuados de actividad física y el bienestar mental de los individuos, debido a la liberación de neurotransmisores que favorecen esta sensación^9^.

Así mismo, gran parte de los adultos mayores atraviesan esta etapa de sus vidas en soledad, debido al alejamiento de los hijos, el fallecimiento del cónyuge o el abandono familiar, las relaciones interpersonales también disminuyen por el proceso de jubilación, la comorbilidad y la ausencia de motivaciones, unido a éstas la ínfima inclusión en los programas estatales, son situaciones que convergen en el empeoramiento de su salud, en una paupérrima calidad de vida^10^, ineficiente capacidad de resiliencia y en una reducción importante de su esperanza de vida^11^.

A pesar de ello, el sistema de salud peruano ha descuidado la salud de las personas de la tercera edad, no garantiza un envejecimiento saludable, y las intervenciones que se realizan no logran cubrir las demandas y necesidades de esta población, el modelo de atención actual no contribuye eficazmente al trabajo preventivo-promocional, pues las intervenciones son mayormente recuperativas con resultados parcialmente óptimos, por la logística precaria en el primer nivel de atención^12^; por tanto, ante estas circunstancias el rol de los profesionales de la salud adquiere una importancia notable, para brindar las pautas y el acompañamiento progresivo en el manejo global de las patologías y sus complicaciones^2^. Así mismo, es necesario que las políticas sean integrales y se focalicen según las características de cada contexto sociodemográfico en el que residen los adultos mayores^13^, para abarcar a los menos favorecidos por su condición económica precaria^14^.

En este sentido, lo más valioso para lograr un envejecimiento saludable y satisfactorio en los adultos mayores es que las intervenciones se enfoquen tempranamente en la prevención de las enfermedades y la promoción de estilos de vida saludables, metas que solo se podrán lograr con el trabajo arduo y el compromiso serio, sensible y sostenible de quienes formulan las políticas de salud, por lo pronto el desarrollo de investigaciones que muestren el panorama actual y futuro de los adultos mayores constituyen un aporte preponderante.

La fragilidad de los adultos mayores y su endeble inclusión en el sistema de salud, resaltaron la importancia de la investigación, que permitió plantear como objetivo: establecer el diagnóstico clínico integral según sexo y edad de adultos mayores atendidos a nivel nacional en el Perú, en tanto que la hipótesis formulada fue: el diagnóstico clínico integral según sexo y edad de adultos mayores atendidos a nivel nacional en el Perú, presenta diferencias estadísticamente significativas

## Materiales y Métodos

Se realizó un estudio descriptivo, transversal y retrospectivo, durante mayo y junio del 2021. La población estudiada fue de 60698 adultos mayores de 60 años a más, se incluyeron usuarios de ambos sexos y de todos los departamentos del Perú, atendidos en los establecimientos de salud del Ministerio de Salud durante enero y septiembre del 2017. Se excluyeron a los usuarios con información incompleta.

El diagnóstico clínico integral fue establecido por el Ministerio de Salud mediante la Guía Técnica para el llenado de la Historia Clínica de Atención Integral de Salud del Adulto Mayor^15^, en las siguientes categorías:

*Adulto mayor enfermo.* Aquellos que presentan patologías agudas o crónicas no invalidantes.
*Adulto mayor frágil.* Los que presentan dos o más de las siguientes características: edad de 80 años a más, dependencia parcial en la valoración funcional, deterioro cognitivo leve o moderado, manifestaciones depresivas, riesgo social, caídas en el último mes o más de una caída en el año, tres o más patologías crónicas, patología crónica que provoca incapacidad funcional parcial, consume más de tres fármacos para patologías crónicas, hospitalización en el último año o índice de masa corporal igual o menor a 23 kg/m2 o mayor a 28 kg/m2.
*Adulto mayor geriátrico complejo.* Los que presentan tres o más de las siguientes características: edad de 80 años a más, tres o más patologías crónicas, patologías incapacitantes, deterioro cognitivo severo, problema social o paciente en etapa terminal.
*Adulto mayor saludable.* Los que tienen características físicas, funcionales, mentales y sociales acorde a su edad, con ausencia de patologías.

Los datos de las variables analizadas se obtuvieron de la base de datos abiertos del Ministerio de Salud del Perú^15^ disponibles en un archivo de valores separados por comas de Microsoft Excel. La información contenida en el archivo corresponde a: año, mes, ubigeo, departamento, provincia, distrito, edad (60 a 69 años, 70 a 79 años y 80 a más años), sexo, diagnóstico clínico del adulto mayor y número de casos. La información fue exportada a un archivo de hoja de cálculo de Microsoft Excel, para ordenarla y organizarla.

Se describieron frecuencias absolutas y relativas para las variables analizadas, se estimaron intervalos de confianza para proporciones al 95% y se realizó el análisis bivariado mediante el chi cuadrado de homogeneidad, con una significancia estadística de p < 0,05. El análisis estadístico se desarrolló en el programa estadístico SPSS versión 26 para Windows, la matriz de datos está disponible en Mendeley Data^16^.

Los principios éticos se respetaron y aplicaron en todo el proceso investigativo, así mismo la base de datos no contiene información personal identificatoria de los usuarios, lo que garantiza la privacidad y su anonimato. Los datos se recolectaron de fuentes secundarias, en consecuencia, el investigador no tuvo contacto con los sujetos de estudio.

## Resultados

Se estudiaron a 60698 adultos mayores. Según el diagnóstico clínico integral el 49,4% de adultos mayores estaban enfermos, por sexo, el 50,8% fueron mujeres y el 47,4% varones, el diagnóstico clínico por sexo presentó diferencias estadísticas significativas (p = 0,000) (Tabla 1). Vale decir que la mitad de adultos mayores padecen de al menos una enfermedad aguda o crónica, independientemente del sexo, que les impide de manera parcial cumplir con el desarrollo de sus actividades diarias.

**Tabla 1:** Diagnóstico clínico integral según sexo de los adultos mayores

Diagnóstico clínico	Sexo				Total	
	Femenino (35985)		Masculino (24713)		(60698)	
	n (%)	IC 95%	n (%)	IC 95%	n (%)	IC 95%
AM Enfermo	18285	50,3-51,3	11724	46,8-48,0	30009 (49,4)	49,0-49,8
	(50,8)		(47,4)			
AM Frágil	4666	12,7-13,3	2863	11,2-12,0	7529 (12,4)	12,1-12,7
	(13,0)		(11,6)			
AM Geriátrico complejo	347(1,0)	0,9-1,1	172 (0,7)	0,6-0,8	519(0,9)	0,8-1,0
AM Saludable	12687	34,8-35,8	9954	39,7-40,9	22641 (37,3)	36,9-37,7
	(35,3)		(40,3)			

Del 49,4% de adultos mayores enfermos, el 50,0% tenían de 60 a 69 años, el 50,6% de 70 a 79 años y el 46,1% de 80 a más años, el diagnóstico clínico por edad presentó diferencias estadísticas significativas (p = 0,000) (Tabla 2). Conforme avanza la edad del adulto mayor, la propensión a padecer de enfermedades crónicas se incrementa, debido a sus características intrínsecas y a la vulnerabilidad frente a factores externos.

**Tabla 2:** Diagnóstico clínico integral según edad de los adultos mayores

	Edad						Total	
Diagnóstico clínico	60 a 69 años(22566)		70 a 79 años(25697)		80 a más años(12435)		(60698)	
	n (%)	IC 95%	n (%)	IC 95%	n (%)	IC 95%	n (%)	IC 95%
AM Enfermo	11278(50,0)	50,1-51,5	12993(50,6)	50,0-51,2	5738(46,1)	45,2-47,0	30009(49,4)	49,0-49,8
AM Frágil	1746(7,7)	7,4-8,0	2963(11,5)	11,1-11,9	2820(22,7)	22,0-23,4	7529(12,4)	12,1-12,7
AM Geriátrico complejo	107(0,5)	0,4-0,6	156(0,6)	0,5-0,7	256(2,1)	1,8-2,4	519(0,9)	0,8-1,0
AM Saludable	9435(41,8)	41,2-42,4	9585(37,3)	36,7-37,9	3621(29,1)	28,3-29,9	22641(37,3)	36,9-37,7

## Discusión

Los resultados del estudio presentan similitud con otros desarrollados en contextos diversos. Yao y cols.^5^ en China, encontraron cifras cercanas a la mitad de adultos mayores enfermos (42,4%), los que padecían varias enfermedades de forma simultánea, siendo más frecuente en mujeres (p < 0,05), la diferencia con este estudio es el número de participantes (n = 19841) y la edad que consideraron (> a 50 años). En la India Kshatri y cols.^6^ reportan que alrededor de la mitad de adultos mayores de 60 años a más estaban enfermos (48,8%) y presentaban multimorbilidad, ligeramente superior en mujeres (50,4%); en comparación con el estudio, la cantidad de participantes si fue bastante inferior (n = 725). Del mismo modo, Yadav y cols.^7^ en Nepal manifiestan que el 48,9% de adultos mayores padecían al menos de una enfermedad crónica, siendo las mujeres las más afectadas (52,4%) y los que tenían entre 70 a 79 años (p < 0,05), al igual que el anterior su muestra fue de 794. Las estadísticas de morbilidad y sus características de los adultos mayores del continente asiático no distan mucho con la realidad peruana, pues las características de vulnerabilidad que acompañan al proceso de envejecimiento son propias de la edad y no distingue ámbitos geográficos.

Así mismo, Melo y Lima^8^ en Brasil encontraron una frecuencia de adultos mayores enfermos superior al estudio (53,1%), acentuada en mujeres (p < 0,001) y en edades avanzadas (p < 0,002), las que presentaban de tres a más enfermedades crónicas, el número de participantes fue inferior (n = 11697) al estudio. Independientemente del número de participantes en todos los contextos estudiados, al menos una enfermedad crónica coexiste medianamente en los adultos mayores, sin ser determinante el lugar donde viven, pues el proceso de envejecimiento solo puede ser distinto en su velocidad de progresión y en las condiciones en que se presenta, ya que es una etapa por la que todos los individuos tienen que transitar.

Las enfermedades crónicas se han instaurado en la población adulta mayor y al menos uno de cada dos presenta un cuadro patológico que interfiere en el desarrollo normal de sus actividades y en su calidad de vida, son el resultado de años acumulados de prácticas dañinas para el organismo, exigencias al límite en el funcionamiento corporal, ausencia de chequeos fisiológicos para detección oportuna de alteraciones, y cuando éstas son identificadas son pocos los individuos que asumen con responsabilidad y disciplina los cambios que deben realizar, por el contrario muchos de ellos continúan con prácticas que entorpecen las medidas terapéuticas^17^. Ante esto una de las medidas prioritarias del sector salud debe ser el amplio trabajo de sensibilización de la población, para frenar comportamientos insalubres y factores de riesgo, dado que la carga de estas enfermedades no solo afecta al individuo, sino también involucra a la familia y sociedad^18^.

La forma de presentación de las enfermedades crónicas generalmente es en asociación con otras, dando lugar a la multimorbilidad, y en consecuencia a la polifarmacia, lo que dificulta el manejo individualizado de los pacientes, más aún cuando se tienen protocolos de atención generales en el sistema de salud. Lo peligroso de la multimorbilidad es que altera las condiciones físicas, funcionales, mentales y sociales de los individuos, provocando limitaciones en el esfuerzo físico, alimentación, actividades diarias habituales y en las relaciones interpersonales, según van apareciendo las complicaciones^2^^,^^9^^,^^3^. En este sentido, el trabajo de los profesionales de la salud debe ser multidisciplinario e interdisciplinario.

Las enfermedades presentes en los adultos mayores afectan con mayor frecuencia a las mujeres, esto se debería principalmente al tipo de enfermedades prevalentes por sexo y a su mayor esperanza de vida, siendo las enfermedades cardiovasculares y el cáncer las que lideran la lista, debido probablemente a la exposición temprana en etapas previas al tabaquismo, sedentarismo y mala alimentación^19^, así como a los desequilibrios hormonales^20^. Por otra parte, entre las enfermedades que afectan a los varones están las enfermedades de la piel^21^, deterioro cognitivo^22^, enfermedades del colon^23^ y su afectación ocurre en edades más tempranas. Evidencias que deben tenerse en cuenta a la hora de planificar intervenciones efectivas y sostenibles para el control de los factores de riesgo en la población, dado que las características patológicas por sexo son diferenciales.

Así mismo, conforme avanza la edad de los adultos mayores, se introducen en un proceso de envejecimiento inflamatorio producto de la acumulación descontrolada de radicales libres, activación leucocitaria, de células endoteliales y vasculares, que desencadenan problemas ateroscleróticos y de reactividad plaquetaria. Proceso que, unido a la multimorbilidad y condiciones conexas, incrementan considerablemente el nivel de fragilidad de estas personas, la ocurrencia de discapacidades y la demanda de los servicios de salud^24^, los cuales no están preparados logísticamente para tales fines^12^.

Por todo esto, es más que necesario la reorientación de las políticas de salud enfocándose a un trabajo más inclusivo que acoja integralmente a los adultos mayores, para ofrecerles un envejecimiento de calidad y de autorrealización, así mismo es esencial que este trabajo sea coordinado y planificado para frenar oportunamente el avance de las enfermedades crónicas y evitar el desarrollo de complicaciones, teniendo como aliados clave a la familia y al entorno cercano, por ser los que comparten comportamientos y estilos de vida comunes. Los resultados serán más satisfactorios, si las actividades de prevención y promoción se introducen de manera rigurosa desde etapas previas, mediante una sensibilización y compromiso comunitario masivo, pues es la vía más indicada para alcanzar un envejecimiento saludable^25^.

La información de las variables analizadas se obtuvo de una base de datos digital, lo que constituye la principal limitación del estudio, no obstante, la cantidad de usuarios evaluados proporciona una valoración clínica integral a nivel nacional de los adultos mayores. El reporte de resultados de manera global por parte del sector salud, limita realizar una valoración física, funcional, mental y sociofamiliar específicas según los indicadores puntuales para cada una de las variables, lo que podría favorecer en el planteamiento de propuestas de mejora. Así mismo, el subregistro que existe en diferentes regiones del país, no permite realizar una estimación basada en la realidad y completa de los adultos mayores que son atendidos en los establecimientos de salud, por lo que se sugiere una mejor gestión en el sistema de control de las atenciones para saldar adecuadamente estos impases.

## Conclusión

Se concluye que los adultos mayores en su mayoría estaban enfermos, es decir presentaban patologías agudas o crónicas no invalidantes, siendo estas últimas las más frecuentes por su edad, fueron mujeres y tenían entre 70 a 79 años, el diagnóstico clínico integral por sexo y edad presentó diferencias estadísticamente significativas. Este perfil identificado en el adulto mayor, proporciona una línea de base sobre la cual los dadores de políticas de salud, deberían reformular las estrategias que no están siendo efectivas, operativizar aquellas que no se cumplen en la práctica o plantear nuevas estrategias que se adecúen a la realidad vivida en cada contexto de los adultos mayores.

Los resultados muestran a este grupo etario como el más vulnerable en algún aspecto de su salud, por ende, es necesario que las actividades del sector salud se fortalezcan y focalicen en esta población e incluso en etapas anteriores, con la finalidad de promover un envejecimiento saludable que les permita disfrutar de los últimos años de su vida con calidad, luego de su contribución familiar, laboral, económica y social para el desarrollo del país. Siendo así, recién se estaría cumpliendo con el abordaje de una política en salud inclusiva, que sin distingos es capaz de llegar a todos los individuos en su conjunto, para satisfacer sus demandas en salud.
